# An In Vitro Study of Local Oxygen Therapy as Adjunctive Antimicrobial Therapeutic Option for Patients with Periodontitis

**DOI:** 10.3390/antibiotics12060990

**Published:** 2023-05-31

**Authors:** Lena Katharina Müller-Heupt, Anja Eckelt, John Eckelt, Jonathan Groß, Till Opatz, Nadine Kommerein

**Affiliations:** 1 Department of Oral and Maxillofacial Surgery, University Medical Center Mainz, Augustusplatz 2, 55131 Mainz, Germany; 2 WEE-Solve GmbH, Auf der Burg 6, 55130 Mainz, Germany; 3 Department of Chemistry, Johannes Gutenberg University, Duesbergweg 10-14, 55128 Mainz, Germany; 4 Department of Prosthetic Dentistry and Biomedical Materials Science, Hannover Medical School, Carl-Neuberg-Straße 1, 30625 Hannover, Germany

**Keywords:** antimicrobial, calcium peroxide, oxygen, periodontitis, peroxide, *Porphyromonas gingivalis*, *Streptococcus oralis*

## Abstract

Periodontitis is a common global disease caused by bacterial dysbiosis leading to tissue destruction, and it is strongly associated with anaerobic bacterial colonization. Therapeutic strategies such as oxygen therapy have been developed to positively influence the dysbiotic microbiota, and the use of oxygen-releasing substances may offer an added benefit of avoiding systemic effects commonly associated with antibiotics taken orally or hyperbaric oxygen therapy. Therefore, the oxygen release of calcium peroxide (CaO_2_) was measured using a dissolved oxygen meter, and CaO_2_ solutions were prepared by dissolving autoclaved CaO_2_ in sterile filtered and deionized water. The effects of CaO_2_ on planktonic bacterial growth and metabolic activity, as well as on biofilms of *Streptococcus oralis* and *Porphyromonas gingivalis*, were investigated through experiments conducted under anaerobic conditions. The objective of this study was to investigate the potential of CaO_2_ as an antimicrobial agent for the treatment of periodontitis. Results showed that CaO_2_ selectively inhibited the growth and viability of *P. gingivalis* (*p* < 0.001) but had little effect on *S. oralis* (*p* < 0.01), indicating that CaO_2_ has the potential to selectively affect both planktonic bacteria and mono-species biofilms of *P. gingivalis*. The results of this study suggest that CaO_2_ could be a promising antimicrobial agent with selective activity for the treatment of periodontitis.

## 1. Introduction

Periodontitis is a chronic inflammatory disease that affects the supporting tissues of the teeth, including gums, periodontal ligament, and alveolar bone. It is initiated by bacterial dysbiosis subsequently leading to the formation of pathogenic bacterial biofilms, and thus leading to loss of the tooth-surrounding tissues and tooth [1]. It is strongly associated with anaerobic bacterial colonization and represents one of the most common global diseases [2,3]. 

An important component of the pathogenesis of periodontitis is the ongoing loss of attachment of the tooth-surrounding tissues, which is accompanied by a reduction of the extracellular matrix [4]. Due to its hypoxic environment, the resulting periodontal pocket provides a microbiological niche for anaerobic microorganisms and thus, facilitates a dysbiotic shift of the subgingival microbiota [5]. Within the periodontal pocket, growth of both, facultative anaerobic bacteria, such as *Aggregatibacter actinomycetemcomitans*, and obligate anaerobes, such as *Porphyromonas gingivalis, Tannerella forsythia, Treponema denticola,* and *Fusobacterium nucleatum* is supported [6]. According to the keystone-pathogen hypothesis, those microbial pathogens orchestrate inflammation in the tooth-surrounding tissues [7]. Furthermore, it was shown that the immunological host response is dysregulated in patients at increased risk for periodontitis [2].

The local inflammation provides a particularly nutrient-rich environment for the above-mentioned bacteria. For instance, *P. gingivalis* metabolizes heme and iron, substrates which are abundant in inflammatory tissues due to a consequent increase in vascularization and sulcus fluid flow rate [8].

Therapeutic strategies such as oral hygiene measures combined with surgical and non-surgical periodontitis therapy center upon the reduction of the total oral microbial load [9,10]. However, while subgingival instrumentation is widely regarded as the gold standard treatment for periodontitis [11], it primarily focuses on mechanically disrupting the biofilm without inducing a significant alteration in the saliva microbial composition [6,12]. Analysis of bacterial communities through 16S rRNA gene sequencing revealed that subgingival instrumentation failed to modify bacterial communities in unresponsive sites [13]. Therefore, various approaches have been developed and tested to positively influence the dysbiotic microbiota in the periodontal tissues. For example, probiotics, parabiotics and postbiotics as an adjunct to non-surgical periodontal therapy treatment seem beneficial on specific periodontopathogens [14,15,16].

Oxygen impairs the growth of anaerobic bacteria [17] and thus, oxygen therapy is another approach of adjunct periodontitis therapy. A clinical study by Signoretto et al., indicated that the combination of hyperbaric oxygen therapy (HBOT) and subgingival instrumentation significantly reduced the Gram-negative anaerobic load of the subgingival microflora (by up to 99.9%) and low levels of pathogens persisted for at least two months after therapy. HBOT or subgingival instrumentation alone had a transiently lower effect on periodontal anaerobes and it was also shown that HBOT, both alone and in combination with scaling and root planing, reduced the gingival index score to zero and maintained periodontal health for at least three months [18]. 

In a clinical study by Burcea et al. [19] patients in the HBOT group had significantly better oral health index (OHI-S), sulcus bleeding index (SBI), dental mobility, and periodontal probing depth scores than patients in the control group (subgingival instrumentation only). In a study by Chen et al. [20] HBOT inhibited the growth of subgingival obligate anaerobes and facultative anaerobes as well as *Bacteroides melaninogenicus*, thus promoting periodontal healing.

Since HBOT takes place in a pressure chamber, it is difficult to integrate in the daily dental practice. Therefore, approaches to apply gaseous oxygen or ozone exist and topical oxygen therapy maybe an interesting option. Schlagenhauf et al. [21] repeatedly applied subgingival gaseous oxygen irrigation in previously untreated periodontal patients. They concluded that repeated oxygen insufflations resulted in a significant clinical improvement in baseline periodontal conditions superior to that of the control group. 

The commonly used periodontitis therapy, such as the “van Winkelhoff cocktail”, which combines amoxicillin and metronidazole, effectively targets both anaerobic pathogens and commensal bacteria. Amoxicillin provides a broad coverage against a wide range of gram-positive bacteria, and it also offers additional coverage against gram-negative bacteria [22], whereas metronidazole is frequently prescribed as an antibiotic for treating infections caused by anaerobic bacteria and certain protozoa [23]. This widely employed treatment of amoxicillin-metronidazole has shown superior clinical outcomes, as measured by a reduction in pocket probing depths, compared to a placebo, but due to the challenges associated with prior antimicrobial testing or its feasibility, systemic antibiotics are frequently prescribed empirically to minimize the presence of periodontopathogenic microflora [24]. 

Furthermore, the adjunctive use of local and systemic antibiotic treatment in periodontitis patients resulted in a temporary increase in antibiotic resistance among subgingival microorganisms [25]. Among the antimicrobials examined in a recent review, ampicillin was found to have the highest frequency of resistance (39.5%) in microorganisms present in periodontal diseases, while ciprofloxacin demonstrated the lowest frequency of resistance (3.4%) [26].

The use of oxygen releasing substances could possibly reverse the dysbiosis without destroying too much of the commensal flora. Potentially the use of these substances locally may offer an added benefit of avoiding systemic effects, which are commonly associated with antibiotics taken orally. For this reason, we tested the inhibitory effects of oxygen released by calcium peroxide (CaO_2_) on a commensal bacterium *Streptococcus oralis* and on the periodontopathogen *Porphyromonas gingivalis* both during planktonic growth and on biofilms. 

## 2. Results

### 2.1. Oxygen Release Measurements

**Figure 1:**
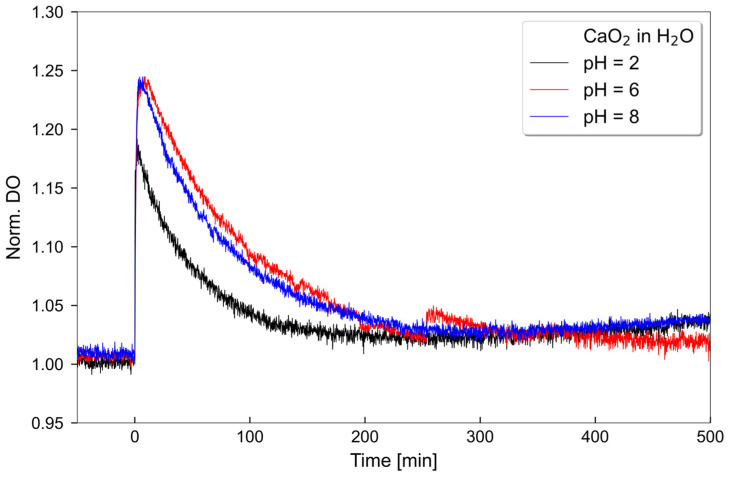
Oxygen release of CaO_2_ at pH 2, 6 and 8. 50 mg CaO_2_ were solved in 5 mL aqua (H_2_O).

The oxygen release behavior of CaO_2_ was evaluated at pH 2, 6 and 8 (Figure 1). Different pH levels were chosen, since pH readings ranging from 2–9 have been observed in periodontal pockets [27]. A decreased amount of available oxygen was observed at pH 2 compared to pH 6 and 8. Despite the potential existence of different pH ranges in periodontal pockets, the investigated areas guarantee a reliable source of oxygen release.

### 2.2. Selective Antimicrobial Effects of Oxygen on Planktonic S. oralis and P. gingivalis

**Figure 2:**
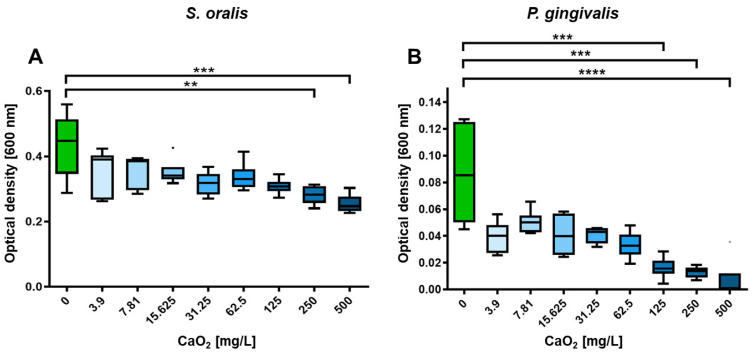
Influence of CaO_2_ on the optical density of planktonic oral bacteria. (**A**) The optical density of the commensal *S. oralis*, decreased significantly at CaO_2_ concentrations of 250 mg/L and 500 mg/L compared to the control. (**B**) The optical density of the periodontitis-associated bacterium *P. gingivalis* significantly decreased at CaO_2_ concentrations of 125 mg/L, 250 mg/L, and 500 mg/L compared to the control. N = 6 with *S. oralis* strain ATCC 9811 and *P. gingivalis* strain ATCC 33277/DSM 20709. Significance level: *p* ≤ 0.05 (** *p* < 0.01, *** *p* < 0.001, **** *p* < 0.0001).

**Figure 3:**
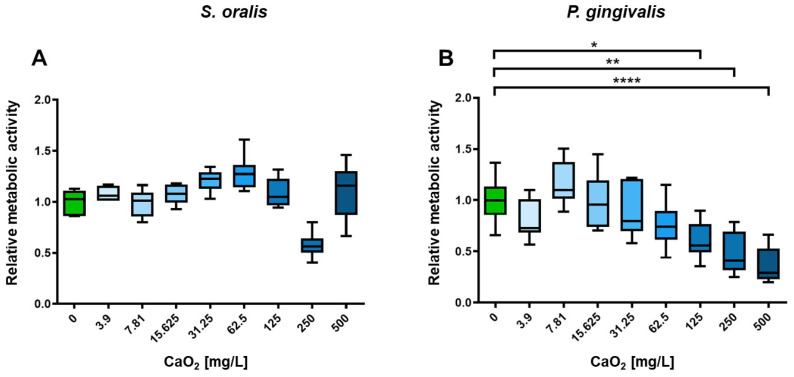
Influence of CaO_2_ on the metabolic activity of planktonic oral bacteria. (**A**) Relative metabolic activity of *S. oralis* showed only some fluctuations but overall was unchanged compared to the control. (**B**) Relative metabolic activity of *P. gingivalis* significantly decreased at CaO_2_ concentrations of 125 mg/L, 250 mg/L, and 500 mg/L compared to the control. N = 6 with *S. oralis* strain ATCC 9811 and *P. gingivalis* strain ATCC 33277/DSM 20709. Significance level: *p* ≤ 0.05 (* *p* < 0.05, ** *p* < 0.01, **** *p* < 0.0001).

To demonstrate the inhibitory efficacy of CaO_2_ on planktonic bacteria, a selection of CaO_2_ concentrations was utilized. Different concentrations of CaO_2_ was added to planktonic bacterial strains *S. oralis* and *P. gingivalis* ranging from 0 mg/L (control) to 500 mg/L. Shown in each case are the optical density at 600 nm, a parameter for the bacterial growth (Figure 2), and the relative metabolic activity (Figure 3). At concentrations above 125 mg/L, the optical density of *P. gingivalis* decreased significantly (*p* < 0.001; Figure 2B), whereas the optical density of *S. oralis* was only significantly inhibited in planktonic growth starting at a concentration of 250 mg/L (*p* < 0.01; Figure 2A).

These results show that a CaO_2_ concentration of 125 mg/L has a statistically significant inhibitory effect on the planktonic growth of the periodontitis-causing bacterium *P. gingivalis*, but not on *S. oralis*, a gram-positive, facultatively anaerobic bacterium of the Streptococcaceae family which is found in the physiological oral flora (Figure 2). Considering the metabolic activity, CaO_2_ shows some fluctuations as little stress response and slightly inhibition by concentrations of 125 mg/L, but no significant influences on *S. oralis*, whereas the metabolic activity of the periodontal pathogenic bacterium *P. gingivalis* is steadily reduced with increasing CaO_2_ concentrations and a significantly influence on the metabolic activity starting from a concentration of 125 mg/L (*p* < 0.05; Figure 3B). The data indicate a selective inhibition of the harmful bacteria, while the physiological flora remains largely unaffected.

### 2.3. Selective Antibiofilm Activity of Oxygen on S. oralis and P. gingivalis

**Figure 4:**
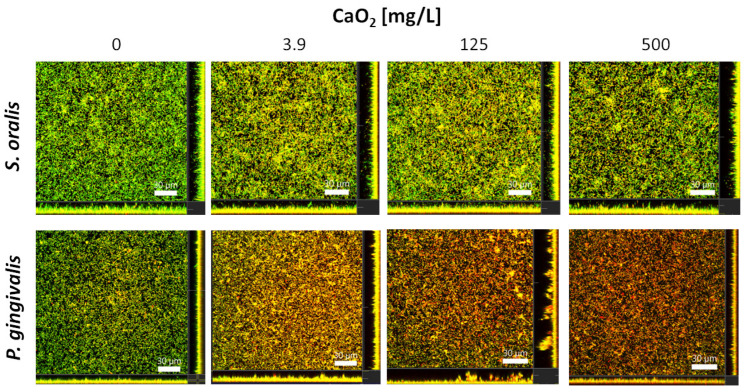
Representative CLSM images of *S. oralis* and *P. gingivalis* biofilms (24 h-old) after two hours treatment with different CaO_2_ concentrations. The large panels display the horizontal sections of the biofilms (x-y planes) and the bottom/side panels show the vertical sections through the biofilms (x-z and y-z planes). The staining of the biofilms was conducted using SYTO 9 and propidium iodide (viable bacteria = green; dead bacteria = red/orange). Scale bars = 30 µm.

**Figure 5:**
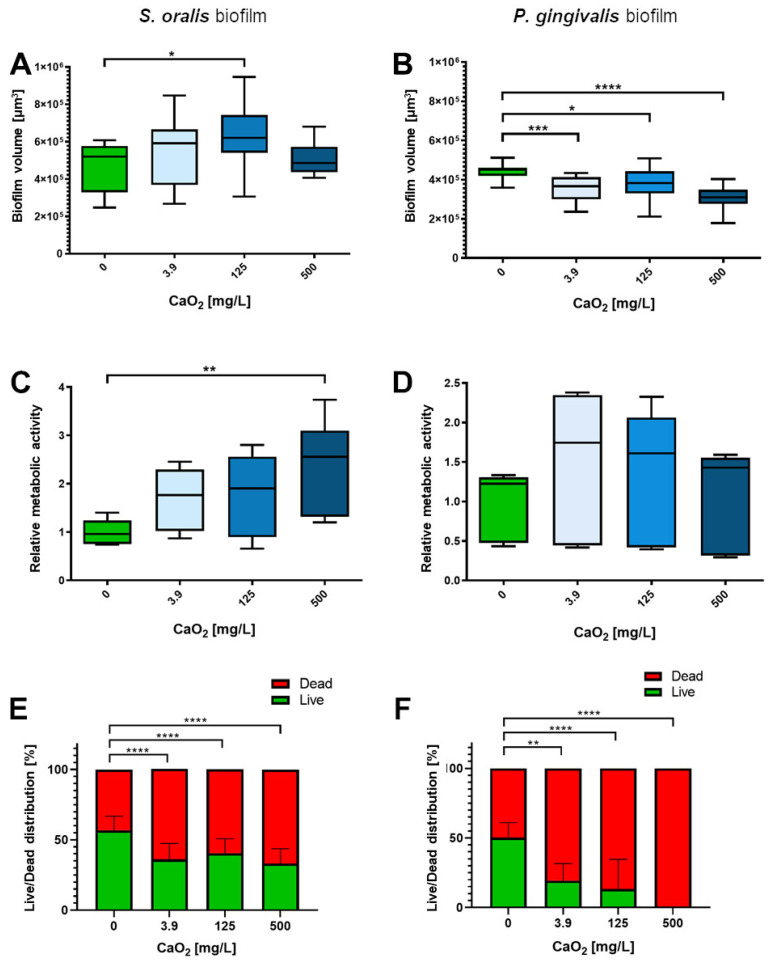
Effect of CaO_2_ on mature *S. oralis* and *P. gingivalis* biofilms after two hours of incubation. (**A**) The biofilm volume [µm^3^] of *S. oralis* increased significantly at a CaO_2_ concentration of 125 mg/L compared to the control. (**B**) The biofilm volume [µm^3^] of *P. gingivalis* decreased significantly at CaO_2_ concentrations of 3.9 mg/L, 125 mg/L, and 500 mg/L compared to the control. (**C**) The metabolic activity of *S. oralis* increased significantly at a CaO_2_ concentration of 500 mg/L compared to the control. (**D**) The metabolic activity of *P. gingivalis* showed no significant difference. (**E**) The percentage of dead bacteria in the *S. oralis* biofilms increased significantly at CaO_2_ concentrations of 3.9 mg/L, 125 mg/L, and 500 mg/L compared to the control. (**F**) The percentage of dead bacteria in *P. gingivalis* biofilms increased significantly at CaO_2_ concentrations of 3.9 mg/L, 125 mg/L, and 500 mg/L compared to the control. N = 6 with *S. oralis* strain ATCC 9811 and *P. gingivalis* strain ATCC 33277/DSM 20709. Significance level: *p* ≤ 0.05 (* *p* < 0.05, ** *p* < 0.01, *** *p* < 0.001, **** *p* < 0.0001).

The proportion of dead bacteria within the *S. oralis* biofilms visually increased slightly to a similar level for each of the three tested CaO_2_ concentrations 3.9, 125 and 500 mg/L after two hours of incubation, whereas the number of dead bacteria in the biofilms of *P. gingivalis* increased more visibly with increasing CaO_2_ concentration, as shown by the representative confocal laser scanning microscope (CLSM) images (Figure 4). The *S. oralis* biofilm volume was significantly higher at a CaO_2_ concentration of 125 mg/L than the volume of the control biofilms (*p* < 0.05; Figure 5A). The metabolic activity enhanced with increasing CaO_2_, significantly at a concentration of 500 mg/L, compared to the control biofilms (*p* < 0.01; Figure 5C). As detectable with the Bacterial Viability Kit, the number of viable bacteria in the *S. oralis* biofilms was reduced by only about 20% compared to the control biofilms (Figure 5E). The biofilm volumes of *P. gingivalis* decreased significantly at all tested CaO_2_ concentrations ranging from 3.8 mg/L (*p* < 0.001) over 125 mg/L (*p* < 0.05) up to 500 mg/L (*p* < 0.0001; Figure 5B). Metabolic activity was highest at 3.9 mg/L and decreased slightly with increasing concentration of CaO_2_ (Figure 5D). The number of viable bacteria within the *P. gingivalis* biofilms decreased significantly to 0% at a concentration of 500 mg/L CaO_2_ (*p* < 0.0001; Figure 5F).

## 3. Discussion

Calcium peroxide is one of the most thermally stable inorganic peroxides and a versatile and effective solid source of hydrogen peroxide [28]. Upon contact with water, calcium peroxide can easily decompose to hydrogen peroxide, hydroperoxide anions or gaseous oxygen [29], which enables the application as an oxygen generator for combating undesired microorganisms. Compared to other metal peroxides, calcium is an essential element for living organisms and the formed carbonates, oxides and hydroxides are also non-toxic [30]. The latter also finds application as a root canal sealant and even show an improved healing pattern between the root and the surrounding tissue after tooth replacement [31,32,33]. Due to the easy use of CaO_2_, its long shelf life, safe transport and thermal stability, it is clearly preferable to an aqueous H_2_O_2_ solution [34].

Calcium peroxide possesses limited solubility in water. When water is present, calcium peroxide slowly dissolves under formation of hydroxide ions, hydrogen peroxide (H_2_O_2_) and hydroperoxide anions (OOH^−^). The latter two species can and will release oxygen gas and calcium hydroxide (Ca(OH)_2_) is “formed”.

Furthermore, numerous experimental studies have demonstrated that calcium ions play a critical role in cellular signalling and can substantially promote wound healing. Calcium-releasing materials have been found to stimulate angiogenesis, collagen and extracellular matrix protein synthesis, and overall tissue granulation [35,36]. The decay of biomaterials containing Ca^2+^ generates a localized, calcium-abundant microenvironment that has been employed as a catalyst for wound healing and/or as an osteoinductive substance. [37,38,39,40]. Moreover, the generation of reactive oxygen species [41,42,43] and their application in antimicrobial dental therapy [44,45,46], as well as the use of CaO_2_ for environmental purposes have been widely reported [47,48,49].

Since the oxygen release rates depend on the pH value of the environment, the dissolved oxygen was measured from CaO_2_ solutions with different pH levels over several hours. The lowest oxygen concentration was found in an acidic environment, while a neutral and alkaline medium possessed the same amount of dissolved oxygen (Figure 1) [50]. pH levels were selected based on the observation of pH readings ranging from 2 to 9 in periodontal pockets [27]. The oxygen release time can be prolonged by dispersing the CaO_2_ particles in a matrix of (bio-) polymers, e.g., hyaluronic acid [51]. 

Anaerobic bacteria are naturally vulnerable to oxygen either by forming excessive reactive oxygen species (ROS) or by interfering with the redox processes necessary for anaerobic physiology [17]. Oxygen may therefore act as selective antimicrobial agent in anaerobic dominated subgingival biofilms in patients with periodontitis and potentially microbial communities are being reshaped under the influence of oxygen. In in vitro experiments it was shown that *P. gingivalis* does not form biofilms in either capnophilic microaerophilic or aerobic conditions [52]. This is in line with the results of our experiments, which indicate a selective inhibition of *P. gingivalis*, while the physiological commensal bacterium *S. oralis* remains largely unaffected. The optical density and metabolic activity of *P. gingivalis* significantly decreased at CaO_2_ concentrations of 125 mg/L. The same concentration did influence neither the optical density nor the metabolic activity of *S. oralis*. Higher concentrations of 250 mg/L and above increased the optical density of *S. oralis*, a gram-positive aerobic oral commensal.

Hypoxia comes with the risk of highly reactive oxygen species (ROS) production [53], which may lead to oxidation of bacterial RNA, DNA, proteins or lipids [54] and thus, high oxygen levels affect aerobic bacterial metabolism as well. ROS are crucial for the host’s defense against microbes and inflammation and elevated levels of ROS facilitate the elimination of invading bacteria [55]. Three naturally occurring ROS species are the superoxide anion (O_2_^−^), hydrogen peroxide (H_2_O_2_), and the hydroxyl radical (HO^•^) [56]. Insufficient levels of ROS in humans can cause recurrent and severe bacterial infections [56], while uncontrolled ROS release can result in excessive inflammation and pathological conditions [57]. 

CaO_2_ may produce ROS as a byproduct during the decomposition of oxygen. The accumulation of hydrogen peroxide (H_2_O_2_) as a residual ROS can significantly increase the generation of free radicals and decrease cell viability. To address this, catalase, an enzyme found in most living cells responsible for breaking down hydrogen peroxide into water and oxygen, can be introduced as an antioxidant [58] or CaO_2_ can be dispersed in alginate or hyaluronic acid scaffolds [51,59]. Although calcium hydroxide is recognized for its high alkalinity, it is generally regarded as biocompatible [60]. Its extensive use in dental and medical fields is well-established. In dentistry, calcium hydroxide finds common application as a root canal medicament, pulp-capping material, or intra-canal dressing [61]. Importantly, studies have shown that calcium hydroxide has no significant impact on stem cells when used at concentrations below 100 mg/mL [62].

The study demonstrated that CaO_2_ had also a selective impact on biofilms. Both bacterial species showed stress reactions in biofilms, but the comparisons of the live-dead staining are particularly exciting: It was observed that the volume of *P. gingivalis* biofilms was significantly lower under all tested CaO_2_ concentrations whereas the *S. oralis* biofilms were more voluminous under the influence of 3.9 and 125 mg/L compared to the control. Therefore, it seems that CaO_2_ triggers an increase in *S. oralis* growth and a reduction, presumably by detaching, in *P. gingivalis* biofilms. Overall, the percentage of viable bacteria in the biofilms was significantly reduced for both species at each concentration tested, but the number of viable bacteria in *S. oralis* biofilms was only reduced by about 20% compared to control biofilms, while the viable fraction in *P. gingivalis* biofilms was reduced to as low as 20% at 125 mg/L and as low as 0% at 500 mg/L CaO_2_.

However, it is important to acknowledge the limitations of this in vitro study. Periodontitis is a complex disease that is strongly associated with dysbiotic polymicrobial biofilms [63]. Apart from *P. gingivalis*, other anaerobic gram-negative bacteria such as *Tannerella forsythia* and *Treponema denticola* play significant roles in the development of this oral disease [64]. Therefore, this study should be regarded as an initial and promising preliminary work. Further research is needed to evaluate the impact of oxygen released by CaO_2_ on other pathogens involved in periodontitis, as well as multi-species periodontal biofilms, both in vitro and in vivo.

For future applications, it is crucial to determine the appropriate type of application (e.g., as a gel) and the optimal duration of CaO_2_ exposure required to achieve the desired targeted antibacterial effect.

In general, it was demonstrated using oxygen microelectrodes that biofilms possess extensive anoxic regions, with oxygen penetrating only about 50 μm into the biofilms that had an average thickness of 210 μm [65]. The relevance of this is highlighted by the fact that the ability of different bacterial species to form biofilms has been demonstrated to be influenced by the availability of oxygen [66]. Furthermore, experimental studies have shown that the efficacy of antibiotics against *Pseudomonas aeruginosa* is reduced under anaerobic conditions [65]. Therefore, local oxygen therapy could positively influence subgingival biofilms and foster eubiosis due to a selective suppression of anaerobic bacteria such as *P. gingivalis*. Nevertheless, it has to be taken into account, that eukaryotic cells of periodontal tissues as well as different bacterial species in subgingival biofilms consume oxygen in vivo.

To foster oral eubiosis, innovative methods that may complement non-surgical periodontal treatment comprise the creation of targeted antimicrobial agents, such as oxygen, plant extracts, probiotics, parabiotics, and postbiotics [14,15,51,67,68]. Additionally, host modulation can serve as an effective supplementary therapy, and combining it with the restoration of oral eubiosis is essential for the development of microbial peptides, antimicrobial peptides, and inflammasome inhibitors [69,70].

## 4. Materials and Methods

### 4.1. Oxygen Release Measurements

50 mg CaO_2_ (75% 200 mesh, Merck, Darmstadt, Germany) was suspended in 5 mL deionized water (millipore grade). The concentration profile of dissolved oxygen was determined by means of a benchtop dissolved oxygen meter HI 5421 (Hanna Instruments, Vöhringen, Germany) as previously described [51].

### 4.2. Bacterial Strains and Culture Conditions 

*S. oralis* strain ATCC 9811 was purchased from the American Type Culture Collection (ATCC) and *P. gingivalis* strain ATCC 33277/DSM 20709 from the German Collection of Microorganisms and Cell Cultures (DSMZ). To obtain 24 h-old precultures for further processing, both bacterial strains were cultured anaerobically (80% N_2_, 10% H_2_, 10% CO_2_; anaerobic workbench Concept 400-M, Ruskinn Technology Ltd., Pencoed, UK) for 24 h at 37 °C in Brain Heart Infusion Medium (BHI; Oxoid, Wesel, Germany) supplemented with 10 µg/mL vitamin K (Roth, Karlsruhe, Germany; BHI/VitK). 

### 4.3. Preparation of Calcium Peroxide Solutions

Autoclaved CaO_2_ (75% 200 mesh, Merck, Darmstadt, Germany) was dissolved in sterile filtered, deionized and autoclaved water. For planktonic experiments, the CaO_2_ was serially diluted with water and for biofilm experiments with BHI/VitK to concentration of 3.9–500 mg/L. For each experiment, each solution was freshly prepared.

### 4.4. In Vitro Investigation of CaO_2_ Effects on Planktonic Bacterial Growth and Metabolic Activity of S. oralis and P. gingivalis

The planktonic experiments were conducted as described in our previous study [68]. Therefore, planktonic *S. oralis* and *P. gingivalis* 24 h-old precultures were adjusted to an optical density of 0.2 (OD_600_; BioPhotometer, Eppendorf, Hamburg, Germany) before they were diluted 1:10 with 2× BHI/VitK to 0.02. By combining bacterial cultures (OD_600_ = 0.02) with newly dispersed CaO_2_ solutions, a final OD_600_ = 0.01 was achieved through equal mixing. The final concentrations of CaO_2_ in the experiments were 3.90, 7.8125, 15.625, 31.25, 62.5, 125, 250, and 500 mg/L.

To include positive controls for bacterial growth, bacterial cultures (OD_600_ = 0.02) were mixed in a 1:2 ratio with sterile, filtered, deionized, and autoclaved water. A negative control (medium sterility) was prepared by mixing 2× BHI/VitK in equal volumes with sterile, filtered, deionized, and autoclaved water. For controls on the sterility of CaO_2_ solution, CaO_2_ dispersions were mixed with 2× BHI/VitK in a 1:2 ratio. Into each well of a 96-well plate (Nucleon 96 Flat Bottom Transparent Polystyrene; Thermo Fisher Scientific, Waltham, MA, USA) 150 µL of the suspensions was pipetted and incubated under anaerobic conditions (Anaerobic Jar and AnaeroGen; Oxoid, Wesel, Germany) with rotation (180 rpm; Shaking Incubator Typ 3032, GFL, Burgwedel, DE) for 24 h at 37 °C. Each experiment was carried out in three biological and two technical replicates.

In order to assess the impact of CaO_2_ on the growth of *S. oralis* and *P. gingivalis* in planktonic cultures, determination of the bacterial growth and metabolic activity was performed as described previously [68]. A plate reader (Tecan, Mennedorf, Switzerland) was used to measure the optical density (OD_600_) of the planktonic cultures directly in the 96-well plates. Furthermore, the BacTiter-Glo^TM^ Microbial Viability Assay (Promega, Mannheim, Germany) was performed for metabolic activity analysis. Therefore, 50 µL of planktonic cultures were transferred into opaque 96-well plates (Nucleon 96 Flat Bottom Black Polystyrene; Thermo Fisher Scientific, Waltham, MA, USA), mixed with 50 µL BacTiter-Glo^TM^ reagent and incubated light-protected and at room temperature for 5 min. Subsequently, the luminescence was measured with the plate reader. All results were normalized to the medium or to the control CaO_2_ dispersion.

### 4.5. In Vitro Investigation of CaO_2_ Effects on S. oralis and P. gingivalis Biofilms

The biofilm experiments were conducted as described in our previous study [68]. Therefore, the OD_600_ of 24 h-old precultures was adjusted to 0.1 using 1× BHI/VitK medium. In each well of a 6-well plate (Cellstar; Greiner Bio-One, Frickenhausen, Germany), 2 mL of each bacterial culture (OD_600_ = 0.1) were added. Following a 24-h initial growth period in anaerobic conditions at 37 °C, the biofilms were subjected to a two-hour treatment with 3.9 mg/L, 125 mg/L or 500 mg/L CaO_2_.

To evaluate the effect of CaO_2_ on biofilms of *S. oralis* and *P. gingivalis*, determination of metabolic activity, biofilm volume and live/dead distribution was carried out as described in our previous study [68]. The metabolic activity of the biofilms was determined using the BacTiter-Glo^TM^ Microbial Viability Assay (Promega, Mannheim, Germany) as follows: After a washing step with Phosphate Buffered Saline (PBS; Biochrom GmbH, Berlin, Germany), the biofilms were rinsed several times with 1 mL of BacTiter-Glo reagent before a light-protected rotating incubation (180 rpm) for 5 min. Subsequently, 100 µL were transferred into opaque 96-well plates in order to measure the luminescence with a plate reader.

For biofilm staining, the LIVE/DEAD BacLight Bacterial Viability Kit (Life Technologies, Carlsbad, CA, USA), containing SYTO 9 and propidium iodide (PI), was performed according to the manufacturer’s recommendations. After staining, the biofilms were fixed for 30 min using 2.5% glutardialdehyde (Carl Roth, Karlsruhe, Germany) in PBS. A confocal laser scanning microscope (CLSM; Leica TCS SP8, Leica Microsystems, Mannheim, Germany) was used for microscopic analysis of the stained biofilms. A laser wavelength of 488 nm was used for SYTO 9 excitation and 552 nm for PI. The *emission* was observed at 500–550 nm (SYTO 9) and at 675–750 nm (PI). Three images were taken of each biofilm with a z-step of 1 μm. The Imaris x64 software (version 8.4.1, Bitplane AG, Zurich, Switzerland) was used for 3D image processing, biofilm volume and live/dead distribution analysis [viable cells = green (SYTO 9); dead cells = red (PI); colocalized cells = orange (SYTO 9 + PI)]. Due to membrane permeabilization, colocalized cells were defined as dead.

### 4.6. Statistical Analysis

GraphPad Prism software 8.4 (GraphPad Software Inc., La Jolla, CA, USA) was used for statistical analysis and graphic processing. Kolmogorov-Smirnov normality test was performed to test for normality. Subsequently, Ordinary One-Way ANOVA with Dunnett’s correction for multiple comparisons (normally distributed data) or Kruskal-Wallis test with Dunn’s correction for multiple comparisons (not normally distributed data) was used to identify statistically significant differences between the samples. The significance level was set to *p* ≤ 0.05 for all comparisons.

## 5. Conclusions

The experiments of this study show that CaO_2_ can have selective effects on the growth and viability of bacterial species—in this case *S. oralis* and *P. gingivalis*—both in planktonic and in biofilm cultures. Further studies should be conducted to assess the influence of oxygen released by CaO_2_ on multi-species periodontal biofilms. 

According to our research, oxygen dissolved from CaO_2_ can effectively hinder the growth of *P. gingivalis*. This suggests that its topical application may be a viable strategy for preventing microbial imbalances caused by the proliferation of harmful bacteria, and thus, halting or slowing the development of periodontitis. Nevertheless, further research may be needed to explore its potential in clinical settings.

## 6. Patents

On 16 February 2022, the patent “Compositions and Kits for the Prevention or Treatment of Gum Diseases” was filed at the European Patent Office in Munich and has received the official file number EP 22 157 021.1.

## Data Availability

Not applicable.
